# Supplementary material for: COVID-19 in Brazilian cities: Impact of social determinants, coverage and quality of primary health care
Source: PLoS One. 2021 Sep 17;16(9):e0257347. doi: 10.1371/journal.pone.0257347 (PMC8448317; doi:10.1371/journal.pone.0257347)
Supplement: Attachment — Submitted filename: Response to Reviewers_Covid_Brazil 20.08.pdf [file pone.0257347.s002.pdf]

FEDERAL UNIVERSITY OF RIO GRANDE DO NORTE

EDITOR
Editor-in-Chief
Plos One

Aug 20, 2021.

Dear Editor,

Thank you for your email with the reviewers' comments. We have reviewed the comments and edited the manuscript accordingly. Please, find attached our point-by-point response to the reviewers. All authors have read this protocol and agreed with Plos One policy. We hope the revised manuscript is now suitable for publication.

Sincerely. Johnnatas Mikael Lopes.

Reviewer Comments:

Comments to the Author:

1. If the authors have adequately addressed your comments raised in a previous round of review and you feel that this manuscript is now acceptable for publication, you may indicate that here to bypass the "Comments to the Author" section, enter your conflict of interest statement in the "Confidential to Editor" section, and submit your "Accept" recommendation.
Reviewer #2: (No Response)

2. Is the manuscript technically sound, and do the data support the conclusions?

The manuscript must describe a technically sound piece of scientific research with data that supports the conclusions. Experiments must have been conducted rigorously, with appropriate controls, replication, and sample sizes. The conclusions must be drawn appropriately based on the data presented.
Reviewer #2: Yes

3. Has the statistical analysis been performed appropriately and rigorously?
Reviewer #2: Yes

4. Have the authors made all data underlying the findings in their manuscript fully available?

The PLOS Data policy requires authors to make all data underlying the findings described in their manuscript fully available without restriction, with rare exception (please refer to the Data Availability Statement in the manuscript PDF file). The data should be provided as part of the manuscript or its supporting information, or deposited to a public repository. For example, in addition to summary statistics, the data points behind means, medians and variance measures should be available. If there are restrictions on publicly sharing data—e.g. participant privacy or use of data from a third party—those must be specified.

Reviewer #2: (No Response)

Response: All data used for this manuscript is in the public domain. In the Methods section you will find all this information, as well as links to access the full material.

5. Is the manuscript presented in an intelligible fashion and written in standard English?

PLOS ONE does not copyedit accepted manuscripts, so the language in submitted articles must be clear, correct, and unambiguous. Any typographical or grammatical errors should be corrected at revision, so please note any specific errors here.
Reviewer #2: Yes

6. Review Comments to the Author

Please use the space provided to explain your answers to the questions above. You may also include additional comments for the author, including concerns about dual publication, research ethics, or publication ethics. (Please upload your review as an attachment if it exceeds 20,000 characters)

Reviewer #2:
Methods –
1. The COVID-19 reporting systems where data were collected were not detailed. Are they mild and serious cases? Were the systems used e sus ve (mild) and sivepripe (severe)?

Response: Thank you for your comments. In the present study, no differentiation was made between severe and mild cases. Cases diagnosed by COVID-19 were used, according to data extracted from the Covid-19 Panel (https://covid.saude.gov.br/) database, fed by the Health Surveillance Department and made available by the SUS Informatics Department. This information can be found in the methods section of the manuscript.

2. The standardization of the incidence coefficient would be important, due to the difference in the impact of the disease in the elderly. The proportion of elderly people (> = 60 years) varies from 6.9% in Boa Vista to 20.4% in Porto Alegre, according to data from DATASUS in 2020.

Response: We agree with the reviewer that the higher proportion of elderly people in the capitals of the southern region of the country make them more likely to have COVID-19 cases. However, this difference in reported proportion does not match official data from the Brazilian Institute of Geography and Statistics, the official body for this information (https://censo2010.ibge.gov.br/sinopse/index.php?uf=43&dados=26#topo_piramide).
In any case, the objective of the research is not to estimate the differences in the load of COVID-19 between the capitals, where standardization would be of great importance, as the age composition would impact the compared estimates.

The objective was to estimate the effects of socioeconomic conditions and health system organization prior to the pandemic on the occurrence of COVID-19 cases. In this facet, the effect of age composition is reduced in the inferences, as the comparison is made with levels of independent variables, such as coverage of the PHC and FHS, which do not suffer a direct effect from the age composition. Furthermore, they assume that the primary health care system must be organized according to the local population profile.
Thus, the inferential analysis does not compare the Brazilian capitals, but analyzes them as a single group, being stratified by factors such as healthcare coverage.

3. Como o estudo foi realizado até a semana 26 de 2020, havia apenas critérios para confirmação dos casos (laboratoriais e clínico-epidemiológicos). (Fonte: arquivo: /// C: /Users/AnaRibeiro/Downloads/GuiaDeVigiEp-final.pdf). Após 05/08/2020 (SE 31), os novos critérios clínicos e de imagem clínica foram incluídos (https://portalarquivos.saude.gov.br/images/af_gvs_coronavirus_6ago20_adjustments-finalis-2.pdf).

Response: Thank you for your comments. The following sentences were added:
To date, only criteria for confirmation of cases (laboratory and clinical-epidemiological) were used as a form of diagnosis of Covid-19, using immunological tests, rapid test or classical serology for the detection of antibodies.

Results.

4. Figure 1 shows the image per city per 100,000 inhabitants per city. What is considered each circle. Which cities are shown.

Response: Figure 1 shows a general graph of the incidence in each city analyzed (circles) in order to show the exponential evolutionary profile in all of them.

5. table 1: I do not understand some results. In situations of PHC coverage below 50%, PMAQ score low demonstrated "nine times more cases" per 100,000 inhabitants COVID-19 cases than those with a score medium or high (B=9.08; p<0.001). In the PHC 50-74% stratum, cities with intermediate FHS coverage and PMAQ scores high showed almost "twice less" (B=2,36) COVID-19 incidence rates than cities with lower ratings.

Response: The first interpretation is correct. This is equivalent to stating that in situations of low PHC coverage, having better quality of care had an impact on the dissemination of COVID-19. On the other hand, the second statement is based on the inverse interpretation of the model's coefficient relative to the worst-case coverage and quality of care. This inversion of interpretation may have led to confusion.

6. For the interpretation of Gini and HDI, it is more appropriate to observe the interaction of these factors and not the main effect. The Gini-HDI interaction shows a negative relationship (B=-72.46; p<0.001), which may suggest a mitigating effect in cities with high HDI and low Gini.

Response: In multivariate analyses, when there is an interaction between factors, their interpretation prevails over that of isolated factors, which may contain measurement biases.

7. PLOS authors have the option to publish the peer review history of their article (what does this mean?). If published, this will include your full peer review and any attached files.

Response: Yes, the authors choose to publish the peer review history of this article

All changes made are highlighted in the manuscript.

Thank you for your comment. The manuscript has been revised accordingly.

Sincerely,

Johnnatas Mikael Lopes
