# Supplementary material for: COVID-19 in Brazilian cities: Impact of social determinants, coverage and quality of primary health care
Source: PLoS One. 2021 Sep 17;16(9):e0257347. doi: 10.1371/journal.pone.0257347 (PMC8448317; doi:10.1371/journal.pone.0257347)
Supplement: Attachment — Submitted filename: Resposta_Revisores 19.06.21.docx [file pone.0257347.s001.docx]

Firstly, we would like to thank the reviewers for their suggestions that would greatly improve the manuscript.

The document was extensively revised and readjusted on several points, as listed in the comments associated with the study specifically below.

Review Comments to the Author

Reviewer #1: The original idea to assess the role of PHC in COVID-19 incidence is good but the adopted methodological approach is very confuse and unclear.

“1. Methods - please cite the original source of data: hospitalizations (SIH), severe cases (SIVEP-Gripe); DATASUS is the "SUS IT department); it is not a dataset.”

*Answer: Text was adequate as suggested.*

“Please expain how the score that assess the Primary Care Teams is calculated. What is evaluated?”

*Answer: Teams are classified as unsatisfactory, poor, regular, good, very good, excellent(9). However, in this study a recategorization was performed in 3 categories: low quality - with categories unsatisfactory and poor put together; medium quality - regular and good put together; and high quality - with very good and excellent teams. The database was access in PMAQ site (*[*http://aps.saude.gov.br/ape/pmaq/ciclo3/*](http://aps.saude.gov.br/ape/pmaq/ciclo3/)*).*

*Text was adequate as suggested.*

“2. What is "social Gini"? Please explain.”

*Answer: The Gini coefficient was used as an instrument to measure the degree of income concentration in the cities, as a measure of social inequality, ranging from 0 to 1, and the closer to 1, the greater the inequality in that location. The calculation of this coefficient is given by the ratio of the areas of the Lorenz curve diagram to the accumulated income of the population. Theses data were collected in the National Household Sample Survey.*

*Text was adequate as suggested.*

“3. COVID-19 incidence rates were adjusted for age?”

*Answer: We agree that age groups have an influence on the occurrence of cases sensitive to the need for health services. However, for the purposes of this study, standardization by age has no implication as the age structures of Brazilian capitals are similar. For example, in all capitals the proportion of people aged 60-64 varies from 1.3% to 2.0% and this pattern will be repeated in other age groups* [*https://censo2010.ibge.gov.br/sinopse/index.php?uf=42&dados=26*](https://censo2010.ibge.gov.br/sinopse/index.php?uf=42&dados=26)*). However, it is to state that the age pattern of contagion is similar among the 27 capitals analyzed according to age.*

*Furthermore, our objective is to identify the effects of ecological, social, economic and health system conditions and not to compare individual conditions that might be affected by a possible distribution discrepancy between age groups.*

“4. Please describe the cities included.

*Answer: The description of the capitals of the federative units (states) of brazil were placed in a tableua in the method: non-changing social characteristics in the time series and variables that changed over time.*

“5. Please describe the number of cases and the rates for each city.”

*Answer: Text was adequate as suggested.*

“6. Figures are not informative and hard to follow.”

*Answer: Text was adequate as suggested.* The four graphics in Figure 1 were submitted separately to facilitate the editorial process. They are arranged from A-D.

“7. Where are the results of the univariate analysis?”

*Answer: They were included in tableau 1 as they are not results directly produced in our research. This information exists in the Brazilian information system. Furthermore, our objective is to evidence the effects of the independent variables described in our method.*

“8. Why authors have decided to include in the multiple model only variables with p<0.10? (too scrict criterium).”

*Answer: Sorry, it was a typo. In fact, the p-value criterion to enter the model was <0.20. Text was adequate as suggested.*

“9. It is not clear to me if the authors intend to evaluate the influence of socioeconomic and PHC variables in the cumulative incidence of COVID-19 until the 26th week in those cities or if the authors wanted to evaluate the influence of socioeconomic and PHC variables in time-trends in incidence of COVID-19 until the 26th week in those cities.
Please clarify.”

*Answer: Data were analyzed using a matrix of autoregressive correlations in the GEE method. This approach allows us to verify the effects of independent factors on the outcome trend. As the trend of cases in the analyzed period is increasing (week factor), factors that influence the trend have implications for the increase in accumulated cases.*

**Reviewer #2**

“Introduction
1. The world has been suffering from a persistent contagion evolution by the new coronavirus (COVID-19 ),
Review: The new virus is SARS-COV-2 and COVID-19 is the disease caused of the virus.”

*Answer:* *Text was adequate as suggested.*

“2. Therefore, the objectives of this study were to identify the socioeconomic and health system factors associated with the diagnosis of COVID-19 in the main cities of each twenty-seven Brazilian federative units, characterizing them in terms of disease burden and evolutionary pattern, providing information for planning public policies for Brazil and countries with a similar profile.
Review: It does not seem to me that the aim of the study was to assess which socioeconomic factors were associated with the diagnosis of COVID-19. Associated factors could be access to health services for symptomatic cases and the number of tests offered.”

*Answer: After the analysis, we adjusted the objective. Therefore, the objectives of this study were to identify the socioeconomic and health system factors associated with the accumulation of cases of COVID-19 diagnosed in the capitals of the twenty-seven Brazilian federative units (FU).*

“Methodology
1.This is an ecological time series study, with quantitative approach, carried out through the analysis of population-based secondary data. The outcome was COVID-19 cases per 100,000 inhabitants, in all Brazilian main largest cities until the 26th epidemiological week. Review: It was not clear the size of the selected cities.”

*Answer: The selected cities are the capitals of the 27 federated states of Brazil. Text was adequated.*

“2. It was not described in the methodology which COVID-19 case definition and database were used. In Brazil, notification of suspected cases of COVID-19 is mandatory in cases of flu like illness-ILI syndrome or severe acute respiratory syndrome-SARS. After notification, cases are investigated for confirmation of COVID-19 using the following criteria: laboratory, clinical, clinical-epidemiological and clinical-image. The databases are e-sus-VE (ILI) and Sivep-Gripe (SARS).”

*Answer: Text was adequate to meet the suggestions.*

“In the assessment of the Primary Care Teams Certification Score, teams are classified as unsatisfactory - those who have not fulfilled the commitments assumed in the adhesion;
Review. It is necessary to describe the items that were used in the assessment of the PMAQ, the Methodological Teams of Primary Care Certification Score.”

*Answer: Text was adequate to meet the suggestions.*


“In addition to these social indicators, data was also collected on the restriction on education (RE), restriction on housing (RH), restriction on sanitation (RS) and restriction on social protection (RSP).
Review: It would be important to describe which database was used to build the RH, RS and RSP indicator.”

*Answer: Text was adequate to meet the suggestions.*

“It could standardize the incidence coefficients of COVID-19, considering the different age structures in cities, we know that COVID-19 is more severe in the elderly population, with an increase in hospitalizations and deaths.”

*Answer: We agree that age groups have an influence on the occurrence of cases sensitive to the need for health services. However, for the purposes of this study, standardization by age has no implication as the age structures of Brazilian capitals are similar. For example, in all capitals the proportion of people aged 60-64 varies from 1.3% to 2.0% and this pattern will be repeated in other age groups* [*https://censo2010.ibge.gov.br/sinopse/index.php?uf=42&dados=26*](https://censo2010.ibge.gov.br/sinopse/index.php?uf=42&dados=26)*). However, it is to state that the age pattern of contagion is similar among the 27 capitals analyzed according to age.*

*Furthermore, our objective is to identify the effects of ecological, social, economic and health system conditions and not to compare individual conditions that might be affected by a possible distribution discrepancy between age groups.*

“Results
1. A total of 857,741 cases were recorded in the Brazilian cities in the analyzed period, with an average of 15,884.09 (±21,604.43), revealing a heterogeneity in the dissemination of cases, seen in figure 1.
Review: Are the cases confirmed or suspected of COVID-19 in Brazil? SARS, flu-like illness both? What is the study period?”

*Answer: These are all confirmed cases. The period refers to the 13th and 26th epidemiological weeks.*

“2. Figure 1. Distribution of the incidence of COVID-19 cases in Brazilian cities up to the 26th epidemiological week. Circles are the observed cases and line is the general trend of progression of the cases
Review:I didn't find figure 1.”

*Answer: It was probably an error in the submission system. It is found at the end of the manuscript.*

“3. In order to estimate the effect of the independent variables and to control the effect of their interactions, an adjusted model presented in Table 1
Review: Describe better results of the model, and how to interpret the results of beta in the incidence of the disease, when the values are greater than 1 or less. It was not clear the description of these results.”

*Answer: Text was adequate to meet the suggestions.*

Discussion:
1. “Review:It would be interesting to include in the discussion articles that corroborate the results. There is talk in Brazil that the Ministry of Health and governments in general

they have not trained and extensively prepared the single health system, especially primary care to track contacts and other policies that could impact the curve of the epidemic.”

*Answer: Text was adequate to meet the suggestions.*
